# Supplementary material for: Self-contrastive weakly supervised learning framework for prognostic prediction using whole slide images
Source: PLOS Digit Health. 2025 Sep 30;4(9):e0000972. doi: 10.1371/journal.pdig.0000972 (PMC12483252; doi:10.1371/journal.pdig.0000972)
Supplement: S1 Table — (PDF) [file pdig.0000972.s001.pdf]

S1. Clinicopathological data classes.

Table 1: **Clinicopathological variable values.**

**Parameter**	**List of Values**
Gender	Male, Female
Age	Current age of the patient, in years
Smoking Status	Non-smoker, Smoker
Grade	Low grade, High grade
Stage	Tis, Ta, T1
Concomitant Carcinoma in Situ	No, Yes
Size	Current size of the primary tumor, in centimeters
Focality of the Tumor	Unifocal, Multifocal
