# Supplementary material for: Self-contrastive weakly supervised learning framework for prognostic prediction using whole slide images
Source: PLOS Digit Health. 2025 Sep 30;4(9):e0000972. doi: 10.1371/journal.pdig.0000972 (PMC12483252; doi:10.1371/journal.pdig.0000972)
Supplement: Attachment — Submitted filename: Response to Reviewers.pdf [file pdig.0000972.s002.pdf]

Paper ID: PDIG-D-24-00096 **RC: *Reviewers' Comment***, AR: Authors' Response, *SC: Suggested Change*

Introduction

Dear reviewers,

We sincerely thank you for your thorough review and valuable feedback on our manuscript. Your constructive comments have greatly helped us improve our work.

We have carefully addressed each of your suggestions and made revisions accordingly. Below, we provide detailed responses to your comment in the following order:

1. (**RC**): This section presents the reviewer's comment exactly as provided.
2. (AR): This section contains the authors' response to the reviewer's comment, addressing the concerns raised.
3. (*SC*): This section details the specific changes that have been implemented in the manuscript based on the reviewer's feedback.

We hope the revised manuscript meets your expectations and is now suitable for publication.

Sincerely,
The authors

Reviewer #1

RC#1: ***I suggest that the fourth line of the paragraph be augmented with a citation to a related paper. This citation would serve to add more context to the nature of weakly labeled data.***

AR#1: We agree with the reviewer that citations better contextualize and motivates further the presented research. Unfortunately, the reviewer does not specify which paragraph is being discussed. After some consideration, we deemed that the reviewer is referring to the fourth line of the first paragraph of the subsection Non-Supervised Learning Methods. More specifically, to the sentence "*Weakly supervised methods emerge as an advantageous approach for prognostic applications as they accommodate the uncertainty and heterogeneity of medical data.*" Given this concern, we have added references to support the statement.

SC#1: Added the following references at the end the fourth line of the first paragraph of the subsection Non-Supervised Learning Methods:

- *Schmidt A, Morales-Alvarez P, Molina R. Probabilistic attention based on gaussian processes for deep multiple instance learning. IEEE Transactions on Neural Networks and Learning Systems; 2023;1–14.*
- *Kapse S, Pati P, Das S, Zhang J, Chen C, Vakalopoulou M, Saltz J, Samaras D, Gupta RR, Prasanna P. SI-MIL: Taming Deep MIL for Self-Interpretability in Gigapixel Histopathology. In: Proceedings of the IEEE/CVF Conference on Computer Vision and Pattern Recognition; 2024, p. 11226-11237.*

RC#2: ***A comprehensive reorganization of the paper is warranted. The Introduction section could hold the Background & Related Work, Data Modalities, and Non-Supervised Learning Methods sections.***

AR#2: We acknowledge that defining a section Background & Related Work might have been unnecessary, and subsections Urinary Bladder Cancer, Data Modalities, and Non-Supervised Learning Methods have been relocated to the Introduction section.

SC#2: Section header for Background & Related Work removed.

RC#3: ***Furthermore, the Dataset and Compliance with Ethical Standards should be relocated to the Method section.***

In many publications, the data material is presented in a separate section, which is our usual practice. However, in response to the reviewer's suggestion, we have integrated the data material into the Methods section as recommended.

SC#3: Dataset section reinstated as a subsection of the Methods section, and Compliance with Ethical Standards subsection reinstated as a subsubsection of Dataset subsection. Added a sentence on the first paragraph of the Methods section: "First, we define the dataset utilized to undergo this study. Then, in the subsequent subsections, we introduce a three-step fully-automated pipeline for ...".

RC#4: ***A reconstruction to facilitate a clearer demarcation between the Method section and the Results, will ultimately enhancing readability.***

AR#3: We thank the reviewer for providing comments that enhance the understanding of our research. Given the suggestion, we added a new subsection at the end of the Methods section to summarize the proposed method and briefly tease the content of the experiment and results sections.

SC#4: Added a new subsection titled Deep Learning Pipeline Method for Prognostics, at the end of the Methods section, with the following text: "Our method integrates an automatic ROI tile extraction algorithm, a self-supervised-trained CNN-based feature extraction module, and a weakly supervised aggregation of visual features for prognostic outcome prediction, as displayed in Fig. 2. Ultimately, these individual blocks of the pipeline sequentially process raw data extracted from NMIBC WSI in order to produce a prediction. In the following sections, we will present the results obtained using our proposed method. We will discuss the performance metrics, comparative analysis with baseline models, and insights gained from the attention mechanisms used in the MIL framework, annotations and clinicopathological data."

RC#5: ***I recommend the inclusion of a flow diagram to illustrate the experimental workflow. This visual aid would provide clarity regarding the model optimization process undertaken.***

AR#4: We agree with the recommendation and we have included a new figure with the experimental workflow undergone.

SC#5: Included a new figure under Prognostic Experiments section illustrating the experimental workflow, with the caption: "Experimental diagram of prognostic experiments on NMIBC WSI outcome prediction. Following a systematic experimental approach, diverse design parameters are progressively set based on validation set results. The order of the experiments carried out follows the order displayed."
The following text was added to the manuscript: "Given the numerous choices for contrastive learning loss, backbone, ROI, and magnification levels, coupled with the computational intensity of learning and inference on gigapixel images, we adopt a systematic experiment approach, as illustrated in Fig. 4."

RC#6: ***Several models showcased in Table 7 exhibited performance metrics comparable to random guess or slightly better. The absence of additional metrics precludes a comprehensive analysis of these outcomes and a detailed comparison between the models. The authors overall didn't include a metric other than AUC for evaluating model performance although other metrics such as accuracy and precision are valuable especially for treatment prognosis which may be the basis for choosing a treatment.***

AR#5: We appreciate the reviewer's feedback regarding the inclusion of additional performance metrics. While we understand that metrics such as accuracy and precision are valuable, we opted to use AUC as our primary metric due to its robustness in evaluating model performance across different threshold settings. Including additional metrics in Table 7 could potentially clutter the table and detract from the clarity of the presented data. Therefore, we opted for adding a justification of our metric of choice.

SC#6: Added the following text in the Experimental Setup initial paragraph: "The results are presented in terms of AUC as our primary metric due to its robustness in evaluating model performance across different threshold settings."

RC#7: ***At line 460 the clinical data fed to the model is ambiguous. The unexpected result raises questions about the nature and the form the clinical data where fed to the model.***

AR#6: We apologize if this aspect has not been clearly explained. We define what clinical variables were used in lines 127-129, and how a one-dimensional vector of clinical parameters was formed and used in lines 273-277. Indeed, we acknowledge that these explanations are not properly linked and appear disconnected. Therefore, first, we included a supporting table as an annex containing the values of the clinical parameters. Second, we explain more thoroughly how the vector with clinical parameters is constructed, and link it to the new table.

Moreover, the discussion of the unexpected results is expanded, contemplating the nature and form of the clinical data fed to the model. It is noteworthy that the most valuable clinical data is derived from the manual visual examination of WSIs (grade, stage, among others), leading to information redundancy and potential overfitting when combining the model's features with the clinical data vector. Also, the model may struggle to effectively combine and leverage the clinical data with the imaging features, and other fusion methods might be explored, as proposed in the discussion of the results.

SC#7: Regarding the nature and form of the clinical data used, a Supporting Information S1 annex has been included to describe the clinical parameters utilized and their range of values. The following text was added in order to improve the cohesion and enhance understanding of the concept: "The clinical vector $\mathbf{h}_{var}$ is created by assigning integer values to clinical parameters based on their real-world values (e.g., 0 for non-smoker, 1 for smoker), see Supporting Information S1 annex. The length of this vector corresponds to the number of variables used." Also, added the following text in the Dataset description: "See Support Information S1 for further details on variable values."

Regarding the discussion of the results with clinical data, the following text was added: "It is noteworthy that the most valuable clinical data is derived from the manual visual examination of WSIs (e.g., grade, stage), leading to information redundancy and potential overfitting when combining the model's features with the clinical data vector. Nevertheless, moving forward, exploring different fusion methods ..."

Reviewer #2

RC#1: ***Limited Comparison with Existing Methods: The paper lacks a thorough comparison with existing prognostic prediction methods, especially those that integrate clinical and histopathological data. A more extensive comparison would provide a better context for evaluating the proposed approach's effectiveness.***

AR#1: We appreciate the reviewer's suggestion regarding a more thorough comparison with existing prognostic prediction methods. While we agree that additional comparisons would be beneficial, we have conducted extensive experimentation within the scope of our study. Specifically, we have evaluated our method across multiple datasets, including both diagnostic and prognostic datasets. We have explored various approaches to feature aggregation and extraction, tested different magnification levels, leveraged the use of clinicopathological data, and compared automatic ROIs with annotated regions. These efforts were designed to rigorously assess our method's performance and effectiveness. Although we acknowledge that comparisons with additional methods could provide further context, we believe the extensive evaluations we performed offer a strong foundation for understanding the capabilities and contributions of our approach. Nevertheless, future research will aim to build upon these findings with broader comparisons as more relevant methods and datasets become available.

SC#1: Added a subsection Limitations and Future Research, for which a text specifically addressing the reviewer's comment states: "A noted area for enhancement is the comparison with existing methods. Although the study has introduced innovative approaches, a broader comparison with current state-of-the-art techniques could provide valuable insights into their relative performance, potential advantages and areas for further improvement."

RC#2: ***Dataset Limitations: The paper should provide more information about the datasets used, including their size, diversity, and representativeness. Additionally, addressing any potential biases or limitations in the datasets would strengthen the validity of the results.***

AR#2: We agree with the reviewer that providing more information about the dataset enhances transparency. Unfortunately, we are currently unable to disclose these details as they are part of ongoing research efforts and need to be preserved for future studies to ensure the integrity of our research data. We hope the reviewer understands our position and that the information provided is sufficient for understanding the presented research. We can, however, confirm that a meticulous data curation process and data split was carried out in an informed and representative manner.

SC#2: The following text was added to the Dataset section: "For either cohort, a comprehensive quality assessment of the visual features present in the WSIs was conducted by expert uropathologists. Additionally, it was confirmed that the clinical data documented in the records were accurately represented in the slides. To address potential biases in the study population, a meticulous data curation process was performed, and a data split was established that considered clinical variables deemed representative by expert uropathologists."

RC#3: ***Validation and Generalization: The paper mentions validation results, but it's unclear how well the proposed method generalizes to new datasets or populations. Providing insights into the model's performance across different datasets or in real-world clinical settings would enhance the paper's applicability and reliability.***

AR#3: We agree with the reviewer that further validation would be valuable in demonstrating the generalizability of our methods. However, to the best of our knowledge, no NMIBC WSI datasets are currently available in the literature, which prevents us from validating our methods on other cohorts. Consequently, we have included a statement acknowledging the lack of available datasets for comparative purposes.

It is also important to note that our proposed solution is tailored to a real-world clinical setting, aiming to predict the clinical outcome and inform treatment decisions for NMIBC patients based on the initial trans urethral resection of bladder tumor (TURBT) as the first observation of the tumor state. This is crucial in routine clinical practice, where urologists must determine treatment strategies and assess the potential risk of a tumor upon initial observation of bladder cancer. We recognize that this aspect has not been emphasized enough in the manuscript, and we proposed some changes in the Abstract, Introduction, Methods and Conclusions.

SC#3: The following text was added to the Abstract section: "... in real-world clinical scenarios."
The following text was added to the Dataset section: "To the best of our knowledge, no NMIBC WSI datasets are currently publicly available."
The following text was added to the second paragraph of the Introduction section: "Additionally, timely interventions can greatly enhance treatment efficacy and overall prognosis, making it essential for clinicians to make informed decisions based on the initial tumor observation."
The following text was added to the fifth paragraph of the Introduction section: " ... in a real-world clinical setting."
The following text was rephrased in the Urinary Bladder Cancer subsection of the Introduction section: "Identifying these patients at the first trans urethral resection of bladder tumour (TURBT) would significantly reduce the recurrence rate and treatment cost, hence contributing to more adequate patient-based treatment strategies."
The following text was added to the first sentence of the first paragraph of the Dataset subsection of the Methods section: "... from an initial TURBT ..."
The following text was added to the the first sentence of the first paragraph of the Conclusions section: "...in a real-world clinical setting, ..."

RC#4: ***Interpretability and Clinical Relevance: While attention-guided interpretability is discussed, the paper could delve deeper into how the model's predictions align with clinical practice and whether the identified regions of interest are clinically meaningful. Providing examples of how the model's predictions could inform clinical decision-making would strengthen the paper's relevance to healthcare practitioners.***

AR#4: We acknowledge that demonstrating a correlation between the model outputs and routine clinical practice, specifically in terms of ROIs considered relevant by pathologists, is currently lacking and would better highlight the model's strengths. Therefore, we created a new figure to compare the model outputs with pathologist annotations, along with a discussion text to elaborate on these comparisons.

SC#4: Included a new figure under the subsection Attention-guided Interpretability of the Prognostic Experiments section, with the following caption: "Heatmap of attention scores over annotated areas. Annotated areas by pathologists are overlaid for comparison, demonstrating the correlation between the model's predictions and clinical annotations."
Also, a text is added in the same subsection for discussion: "The heatmap in Fig.7 highlights the regions where the model assigns high attention scores in comparison to ROIs annotated by pathologists, based on clinical experience and histopathological criteria. The strong correlation between the model's high attention areas and the pathologists' annotations suggests that the model effectively identifies clinically relevant regions, supporting its use in diagnostic processes. By pinpointing critical regions, the model assists pathologists in focusing on the most diagnostically significant areas of a slide, enhancing both its transparency and practical utility in a clinical setting. This is particularly valuable in cases of NMIBC with extensive tissue samples, where manual review can be time-consuming. Comparing the attention disparity between classes, unlike the clear distinction seen at the region level, where BCG-R areas were prominently highlighted, the comparison between BCG-R and BCG-NR at the patch level does not reveal significant differences. However, we noted that, for both classes, the model consistently prioritizes areas annotated as cancerous and invasive over those marked for grading. This observation is encouraging, as advanced stages of the disease are typically associated with poorer outcomes."

RC#5: ***Future Directions: While the paper discusses potential improvements and future directions briefly, a more detailed discussion on the limitations of the proposed approach and avenues for future research would provide valuable insights for the research community.***

AR#5: We agree with the reviewer that sound research benefits from highlighting strengths and weaknesses. Therefore, we have added a new subsection to discuss limitations and potential future avenues of research.

SC#5: Added a new subsection titled Limitations and Future Research under the section Conclusions, with the following text: "Models trained on a single cohort may exhibit limitations in generalizing to images with diverse histological characteristics. An additional constraint is the development and evaluation of models using only in-house cohorts, which raises concerns about their generalizability for broader applications. To address these limitations, future research should focus on developing and validating the proposed methods with external cohorts to confirm their applicability across different populations. Furthermore, evaluating the statistical significance of predictions as a valid factor for risk stratification is crucial.
While histopathological features were prioritized in this study, routine practice often involves integrating additional data modalities, such as sequencing. Incorporating omics and immunohistochemistry into prognostic models will also be essential to align with the comprehensive information available to pathologists for disease management decisions. Additionally, examining how insights from diverse diagnostic models could be integrated to improve the quantitative analysis of disease risk is warranted. Future work should explore the implementation of multi-modal architectures to exploit and integrate these data modalities.
A noted area for enhancement is the comparison with existing methods. Although the study has introduced innovative approaches, a broader comparison with current state-of-the-art techniques could provide valuable insights into their relative performance, potential advantages and areas for further improvement."

RC#6: ***Contradiction: One potential contradictory idea in the paper is the finding that histological features may offer more pertinent information for predicting patient outcomes compared to either clinical data alone or their fusion. This contradicts the common practice of integrating clinical and histopathological data to improve prognostic accuracy. Typically, the medical field emphasizes the importance of considering both clinical and histopathological data for accurate prognosis. However, the paper suggests that histopathological images alone may be sufficient for predicting patient outcomes with higher accuracy. This finding challenges the traditional approach to prognostic prediction and raises questions about the relative importance of different types of data in healthcare decision-making. The paper needs to acknowledge this potential contradiction and provide a thorough discussion of the implications. This could include exploring possible reasons for the discrepancy, such as differences in data quality or the specific characteristics of the study population. Additionally, further research could be recommended to validate the findings and determine the optimal approach for integrating clinical and histopathological data in prognostic prediction.***

AR#6: We apologize if this aspect has not been clearly explained and this aspect has been perceived as contradictory. First, we acknowledge that the description of the nature of the clinical data and how it was formed was lacking. Therefore, we included a supporting table as an annex containing the values of the clinical parameters, as well as explaining more thoroughly how the vector with clinical parameters is constructed.
Second, we agree that the assumption that supporting information (e.g. clinicopathological parameters) might boost model performance. We do address this contradiction in the reviewed version of the manuscript, stating that clinical data should result in higher performance but obtaining the opposite was unexpected. Nevertheless, it is noteworthy that the most valuable clinical data is derived

from the manual visual examination of WSIs (grade, stage, among others), leading to information redundancy and potential overfitting when combining the model's features with the clinical data vector. Moreover, the model may struggle to effectively combine and leverage the clinical data with the imaging features, and other fusion methods might be explored, as proposed in the discussion of the results. Another possible explanation for unexpected results might stem from the lack of normalization when using the clinical data, which may introduce variability and bias into the model. Therefore, the discussion of the contradictory results is expanded, contemplating the nature and form of the clinical data fed to the model.

Finally, concerning the data quality, we conducted a comprehensive quality assessment of the visual features present in the WSIs. Additionally, we ensured that the clinical data documented in the records was accurately represented in the slides. To address potential biases in the study population, we carried out a meticulous data curation process and established a data split that considered clinical variables deemed representative by expert uropathologists. We acknowledge that this information was not included in the original manuscript, and we have now provided additional details in these areas. Unfortunately, we are currently unable to disclose these details as they are part of ongoing research efforts and need to be preserved for future studies to ensure the integrity of our research data. We hope the reviewer understands our position and that the information provided is sufficient for understanding the presented research.

SC#6: *Regarding the nature and form of the clinical data used, a Supporting Information S1 annex has been included to describe the clinical parameters utilized and their range of values. The following text was added in order to improve the cohesion and enhance understanding of the concept: "The clinical vector* $\mathbf{h}_{var}$ *is created by assigning integer values to clinical parameters based on their real-world values (e.g., 0 for non-smoker, 1 for smoker), see Supporting Information S1 annex. The length of this vector corresponds to the number of variables used." Also, added the following text in the Dataset description: "See Support Information S1 for further details on variable values."*

Regarding the discussion of the results with clinical data, the following text was added: "It is noteworthy that the most valuable clinical data is derived from the manual visual examination of WSIs (e.g., grade, stage), leading to information redundancy and potential overfitting when combining the model's features with the clinical data vector. Another possible explanation for unexpected results might stem from the lack of normalization when using the clinical data, which may introduce variability and bias into the model, negatively affecting the learning. Nevertheless, moving forward, exploring different fusion methods ..."

Regarding data quality and biases, the following text was added to the Dataset section: "For either cohort, a comprehensive quality assessment of the visual features present in the WSIs was conducted by expert uropathologists. Additionally, it was confirmed that the clinical data documented in the records were accurately represented in the slides. To address potential biases in the study population, a meticulous data curation process was performed, and a data split was established that considered clinical variables deemed representative by expert uropathologists."

<u>Reviewer #3</u>

RC#1: ***While innovative, the manuscript would benefit from more detailed comparisons with existing deep learning methods (PMID: 36268091)and the reasons for choosing NMIA as well as comparison with other prognostic studies ((PMID: 18367107).***

AR#1: We appreciate the reviewer's suggestion regarding a more thorough comparison with existing prognostic prediction methods. While we agree that additional comparisons would be beneficial, we have conducted extensive experimentation within the scope of our study. Specifically, we have evaluated our method across multiple datasets, including both diagnostic and prognostic datasets. We have explored various approaches to feature aggregation and extraction, tested different magnification levels, leveraged the use of clinicopathological data, and compared automatic ROIs with annotated regions. These efforts were designed to rigorously assess our method's performance and effectiveness. Although we acknowledge that comparisons with additional methods could provide further context, we believe the extensive evaluations we performed offer a strong foundation for understanding the capabilities and contributions of our approach. Nevertheless, future research will aim to build upon these findings with broader comparisons as more relevant methods and datasets become available.
Regarding the two suggested publications, we disagree with their relevance to our study because they fall outside the scope of our research. The first publication (PMID: 36268091) focuses on diagnostics, where risk stratification is based solely on diagnostic features. In contrast, our work aims to predict future events, thus focusing on prognostics. The second publication (PMID: 18367107) is purely clinical, relying solely on clinical data and proposing a flow diagram for managing tumors, but it does not address the prediction of clinical outcomes or incorporate imaging data. Therefore, these references do not align with the prognostic and imaging-focused objectives of our research. Nevertheless, we cite those two publications for further contextualization of the Introduction section.

SC#1: Added a subsection Limitations and Future Research, for which a text specifically addressing the reviewer's comment states: "A noted area for enhancement is the comparison with existing methods. Although the study has introduced innovative approaches, a broader comparison with current state-of-the-art techniques could provide valuable insights into their relative performance, potential advantages and areas for further improvement."
Also, we have added the following citations:

- *Barrios W, Abdollahi B, Goyal M, Song Q, Suriawinata M, Richards R, Ren B, Schned A, Seigne J, Karagas M, and others. Bladder cancer prognosis using deep neural networks and histopathology images. Journal of pathology informatics. 2022;13:100–135.*
- *Black PC, Brown GA, Dinney CPN. The impact of variant histology on the outcome of bladder cancer treated with curative intent. Urologic Oncology: Seminars and Original Investigations. 2009;27(1):3–7.*

The first citation is accompanied by the following text in the subsection Data Modalities in the Introduction section: "Traditionally, imaging has been crucial for diagnostics and risk stratification due to its ability to clearly observe and measure features directly related to the state of the tumor. In contrast, prognostics often lack a direct correlation between observable features and clinical outcomes, making prediction based on imaging alone more challenging. Consequently, prognostic applications ..."
The second citation is placed at the end of this sentence in the subsection Data Modalities in the Introduction section: "However, prognostic applications have primarily relied on clinicopathological information more than on image data."

RC#2: ***Also consider have abbreviation list.***

AR#2: This is a valid observation and we have included an abbreviation list accordingly.

SC#2: Please refer to a newly added section "List of Abbreviations".
