# Supplementary material for: Self-contrastive weakly supervised learning framework for prognostic prediction using whole slide images
Source: PLOS Digit Health. 2025 Sep 30;4(9):e0000972. doi: 10.1371/journal.pdig.0000972 (PMC12483252; doi:10.1371/journal.pdig.0000972)
Supplement: Attachment — Submitted filename: PLoS_Digital_Health_Rebuttal__v2_.pdf [file pdig.0000972.s003.pdf]

Paper ID: PDIG-D-24-00096 **RC: *Reviewers' Comment***, AR: Authors' Response, *SC: Suggested Change*

## Introduction

Dear reviewers,

We sincerely thank you for your thorough review and valuable feedback on our manuscript. Your constructive comments have greatly helped us improve our work.

We have carefully addressed each of your suggestions and made revisions accordingly. Below, we provide detailed responses to your comment in the following order:

1. (**RC**): This section presents the reviewer's comment exactly as provided.
2. (AR): This section contains the authors' response to the reviewer's comment, addressing the concerns raised.
3. (*SC*): This section details the specific changes that have been implemented in the manuscript based on the reviewer's feedback.

We hope the revised manuscript meets your expectations and is now suitable for publication.

Sincerely,
The authors

## Editor

**RC#1:** ***AUC Limitation: Acknowledge that AUC alone is insufficient to fully evaluate performance, especially in imbalanced or clinically sensitive contexts where metrics like precision or sensitivity are critical.***

AR#1: We agree with the editor that AUC, although it shows a generic understanding of the model capabilities, it does not provide enough context for a thorough evaluation of all the aspects of the model. Given this concern, we have added statements to reinforce this limitation.

*SC#1: Added a sentence on the first paragraph of the Experimental Setup section: "However, additional metrics such as sensitivity and specificity provide a more comprehensive evaluation for imbalanced or clinically sensitive applications.". Added a sentence on the new last paragraph of the Limitations and Future Research subsection: "Finally, while our model demonstrates promising results, its current performance is only evaluated using the AUC metric, ..."*

**RC#2:** ***Predictive Performance: Clearly discuss why the model's performance may not yet meet standards for clinical use - comparing table 7 and validation results, particularly in treatment outcome tasks requiring higher reliability.***

AR#2: We acknowledge that the achieved level of performance is far from clinical adoption. Therefore, we address this limitation in several sections of the manuscript.

*SC#2: Added the following sentence in the Abstract: "Altogether, this research serves as an initial investigation on the shortcomings of histopathological image analysis for treatment outcome prediction." Added the following sentence in the Weakly Supervised Aggregation for Treatment Outcome Prediction subsection: "We observe a bias gap between the validation and test results, which hint a lack of generalizability or distinct distributions in between sets. Additionally, it is important to emphasize that models exhibiting this level of performance currently demonstrate limited reliability and remain far from being suitable for real-world clinical implementation." Added a sentence on the Recurrence Prediction subsection: "...particularly when the predictive performance remains insufficient for clinical adoption.". Added the following sentence in the Conclusion section: "...it is important to recognize that performance requirements must be substantially higher to meet the standards necessary for clinical implementation.". Added the following paragraph in the Limitations and Future Research subsection: "Finally, while our model demonstrates promising results, its current performance does not yet meet the required reliability standards for treatment outcome prediction tasks. This comes as a result of the previously mentioned data and technical limitations and the inherent complexity of prognostic outcome prediction.".*

**RC#3:** ***External Validation: Explicitly recognize the lack of external validation as a methodological limitation affecting generalizability.***

AR#3: We acknowledge that the generalizability of the model is compromised when data from a single cohort is utilized. We addressed this concern in a previous review . Nevertheless, we have addressed the limited generalizability of models when data diversity is lacking further, reinforcing the fact that one dataset was used and limited clinical adoption opportunities.

*SC#3: Added the following sentence in the Weakly Supervised Aggregation for Treatment Outcome Prediction subsection: "We observe a bias gap between the validation and test results, which hint a lack of generalizability or distinct distributions in between sets. Additionally, it is important to emphasize that models exhibiting this level of performance currently demonstrate limited reliability and remain far from being suitable for real-world clinical implementation." Added a statement in the Recurrence Prediction subsection: "...developing models based on a single private cohort limits generalizability, ...".*

**RC#4:** ***Single Dataset Use: Emphasize that the study was conducted on a single dataset and ensure this constraint is clearly reflected across the abstract, discussion, conclusions, and tone.***

AR#4: We had addressed this limitation in the previous iteration in the Limitation and Future Research subsection. Nevertheless, we have included additional comments throughout the manuscript to emphasize this aspect.

*SC#4: Added a statement on the abstract: "... for a private data cohort.". Added a statement on the Introduction section: "... from a private data cohort.". Added a sentence on the Recurrence Prediction subsection: "However, as previously discussed in the context of BCG-RP, developing models based on a single private cohort limits generalizability, ..."*

**RC#5:** ***Future Work: Suggest directions to improve multimodal fusion, such as attention mechanisms, GNNs, or late fusion strategies.***

AR#5: We acknowledge that we have suggested few choices for alternatively fuse clinicopathological (tabular) data and images. Therefore, we have included other methods in the results and limitations discussions.

*SC#5: Added a sentence in the Fusing Image and Clinicopathological Data: "Moreover, multimodal fusion models might be a more suitable solution for fusing different data modalities than our proposed framework.". Added a sentence in the Limitation and Future Research subsection: "or distinct fusion strategies ... Moreover, alternative architecture reliant on transformers or graphs could potentially raise the achieved performance.".*

**RC#6:** ***These omissions affect the robustness and generalizability of the findings. It is therefore essential that the limitations of the study are clearly and consistently communicated not only in the abstract, but throughout the manuscript, including the discussion, conclusions, and overall tone. Without this, readers may overinterpret the applicability of the results. That said, we believe the paper can be accepted pending these adjustments, provided that the manuscript clearly acknowledges where it falls short, presents these limitations with transparency, and frames the contribution as a foundation for future work rather than a broadly validated solution.***

AR#6: We appreciate the trust of the editor on our research and manuscript to be published after a minor revision. We have applied changes to overall state and more appropriately frame the limitations of our research.

*SC#6: In order to avoid continuous repetition, please have a look at the suggested changes in previous comments.*
